# Therapeutic Targets and Precision Medicine in COPD: Inflammation, Ion Channels, Both, or Neither?

**DOI:** 10.3390/ijms242417363

**Published:** 2023-12-11

**Authors:** Graeme B. Bolger

**Affiliations:** BZI Pharma LLC, 1500 1st Ave N., Unit 36, Birmingham, AL 35203-1872, USA; graemebolger@BZIpharma.com or gbbolger@uab.edu

**Keywords:** cAMP, phosphodiesterase, PKA, CREB, roflumilast, ensifentrine, CFTR

## Abstract

The development of a wider range of therapeutic options is a key objective in drug discovery for chronic obstructive pulmonary disease (COPD). Fundamental advances in lung biology have the potential to greatly expand the number of therapeutic targets in COPD. The recently reported successful Phase 3 clinical trial of the first biologic agent for COPD, the monoclonal antibody dupilumab, adds additional support to the importance of targeting inflammatory pathways in COPD. However, numerous other cellular mechanisms are important targets in COPD therapeutics, including airway remodeling, the CFTR ion channel, and mucociliary function. Some of these emerging targets can be exploited by the expanded use of existing COPD drugs, such as roflumilast, while targeting others will require the development of novel molecular entities. The identification of additional therapeutic targets and agents has the potential to greatly expand the value of using clinical and biomarker data to classify COPD into specific subsets, each of which can be predictive of an enhanced response to specific subset(s) of targeted therapies. The author reviews established and emerging drug targets in COPD and uses this as a framework to define a novel classification of COPD based on therapeutic targets. This novel classification has the potential to enhance precision medicine in COPD patient care and to accelerate clinical trials and pre-clinical drug discovery efforts.

## 1. Introduction

Chronic obstructive pulmonary disease (COPD) is an extremely common, worldwide disorder defined by chronic respiratory symptoms due to abnormalities of the airways and/or alveoli that cause persistent, often progressive, airflow obstruction [1]. It is characterized by a long-term response to lung injury produced by multiple, interacting environmental triggers (such as tobacco smoke, air pollution, and infections) that engender a profound inflammatory response, leading to irreversible and progressive lung damage. The clinical diagnosis of COPD requires confirmation of persistent airflow obstruction as measured by spirometry. This definition of COPD distinguishes it from other lung disorders, such as asthma, which is characterized by the reversibility of airflow obstruction [1,2], or from restrictive lung disease, in which there is a reduction in lung volume (typically because of a reduction in lung elasticity) without airflow obstruction [3].

Although the reduction of environmental triggers (such as smoking cessation) plays an extremely important role in the symptom burden and long-term outlook of patients with COPD, a substantial fraction of patients with COPD will benefit from pharmacological intervention. Several agents with novel mechanisms of action, as well as new molecular entities that expand older classes of drugs, have become available for patients with COPD in recent years. Successful drug development in COPD has benefitted extensively from a precise knowledge of “druggable” targets in lung tissue, which can then be utilized in molecular and cellular assays for drug discovery.

This review will describe the key therapeutic targets utilized in COPD therapeutics, with a special emphasis on emerging targets and their relation to recent advances in lung biology. For the purposes of this review, therapeutic targets are defined as well-defined molecular entities in lung tissue that are the major mode of action of drugs that have been clearly shown to be of significant patient benefit in COPD. Therefore, drugs acting at these targets must have been clearly validated in phase 2 or later clinical trials in patients with COPD and not just by pre-clinical criteria. Almost all of the drugs acting on the targets described in this review have been validated for both safety and efficacy in at least one, and in many cases multiple, phase 3 trials.

To ensure that this review included all key therapeutic targets, the author identified all clinical trials at www.clinicaltrials.gov that contained the keyword COPD, as of 30 October 2023. This search yielded 480 clinical trials. The author narrowed this search to exclude trials in phase I (which, typically, do not assess efficacy as a primary endpoint) and/or early-stage trials open only at a single center and/or in a single country. All key therapeutic targets identified in this search are described in detail in this review.

After reviewing the biology of these well-defined molecular targets, the review will then rigorously discuss emerging evidence that some familiar COPD drugs may exert much of their therapeutic effect on novel or emerging targets. Finally, it will explore how the increasing availability of novel therapies increases both the feasibility and need for a “precision medicine” approach, where biomarkers are utilized with high predictive value to select the most appropriate therapeutic strategy in any individual patient.

## 2. Well-Defined Drug Targets in COPD Therapeutics

### 2.1. Target 1: The β_2_-Adrenergic Receptor

**Figure 1:**
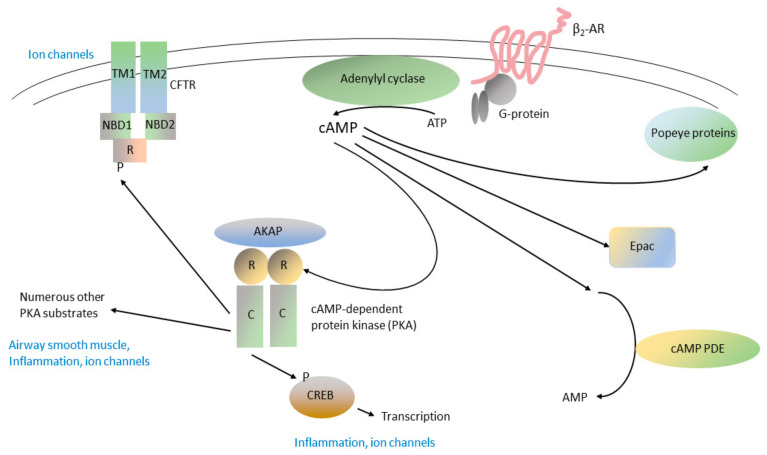
Signal transduction mediated by cAMP in respiratory epithelium: from the β_2_-adrenergic receptor to CFTR and CREB. The β_2_-adrenergic receptor (β_2_-AR) is a member of the G-protein-coupled receptor (GPCR) family, a large family of 7-helix transmembrane proteins that are the physiological receptors for numerous circulating hormones and neurotransmitters and which are also ligands for a myriad of small-molecule drugs, all of which bind to their extracellular or transmembrane regions. GPCRs interact with trimeric G-proteins by recruiting them to specific regions on their intracellular loops. G-proteins have three subunits (α, β, and γ) and the members of the α subunit family can be divided into stimulatory (Gsα) or inhibitory (Giα) isoforms. Agonist binding to the β_2_-AR causes it to interact with, and activate, Gsα. Activated Gsα stimulates various membrane-associated adenylyl cyclase (AC) isoforms (AC6 for the β_2_-AR), which catalyze the synthesis of cAMP. cAMP is a soluble “second messenger” that can diffuse widely in cells but whose concentration is often tightly regulated within specific sub-cellular compartments. As described in the text, cAMP PDEs, notably members of the cAMP-specific PDE, or PDE4 family, selectively hydrolyze cAMP and thereby serve to regulate its levels in cells. The downstream effectors of cAMP include cAMP-dependent protein kinase (PKA), exchange protein activated by cAMP (EPAC), and Popeye proteins. PKA is anchored to specific subcellular locations through its binding to A-kinase anchoring proteins (AKAPs). Binding of cAMP to the PKA regulatory (R) subunits activates the PKA catalytic (C) subunits, which phosphorylate (P) serines and threonines located in clearly defined regions of numerous PKA substrate proteins. A major PKA substrate is the cystic fibrosis transmembrane conductance regulator (CFTR), consisting of a regulatory (R) domain, two nucleotide binding domains (NBD1 and NBD2), and two transmembrane domains (TM1 and TM2), which act as a Cl^−^ ion channel. Phosphorylation by PKA produces a profound change in CFTR R-domain conformation, causing dimerization of NBD1 and NBD2, which in turn activates CFTR Cl^−^ ion channel activity. Another important PKA substrate is the loop-helix-loop transcription factor, cyclic nucleotide response-element binding protein (CREB). Phosphorylation of CREB by PKA and other kinases causes it to dimerize and translocate to the nucleus, where it binds to specific cyclic AMP response elements (CREs) in active chromatin, thereby regulating the transcription of numerous genes. For Figure 1, Figure 2 and Figure 3, the thin curved lines near the top of the figure represent the apical plasma membrane.

The β_2_-adrenergic receptor, one of the best-understood G-protein-coupled receptors (GPCRs), is expressed on pulmonary airway epithelial cells, airway smooth muscle cells, and on specific sub-populations of immune/inflammatory cells in the lung. Inhaled β_2_-adrenergic receptor agonists are separated into two groups based on their pharmacologic half-life: Short-acting β_2_-agonists (SABAs), such as albuterol, are administered every 4 to 6 h (or on an as-needed basis), while long-acting β_2_-agonists (LABAs), such as salmeterol, formoterol, vilanterol, and olodaterol, are administered once or twice daily [4,5,6]. Agonist binding to the β_2_-adrenergic receptor causes it to become engaged with the stimulatory trimeric G-protein G_sα_, which in turn activates membrane-associated adenylyl cyclase (Figure 1; Ref. [7]; see Ref. [8] for a review). Adenylyl cyclase catalyzes the conversion of adenosine triphosphate (ATP) to 3′, 5′ cyclic adenosine monophosphate (cAMP), the prototypical intracellular “second messenger”. Among the most important downstream targets of cAMP is the regulatory (R) subunits of the cAMP-dependent protein kinase (A-kinase, PKA). Binding of cAMP to the PKA R-subunits releases and activates the PKA catalytic (C) subunits, which phosphorylate numerous substrates in cells. In pulmonary epithelium, especially pulmonary ionocytes, which are specialized pulmonary epithelial cells dedicated to the control of ion fluxes [9,10,11], one of the major targets of PKA is the cystic fibrosis transmembrane conductance regulator (CFTR), a Cl^−^ ion channel, which is regulated, at least in part, by the phosphorylation of its regulatory (R) domain by PKA (Figure 1; see section, Target 7, below, for additional details). Another important PKA target is the transcription factor, cyclic AMP response element binding protein (CREB). Other important cAMP targets are the exchange protein activated by cAMP (EPAC) and Popeye proteins [12,13]; their contributions to cAMP signaling in COPD remain a subject of ongoing investigation. All the elements of this pathway are likely to be required for the full range of cellular actions of SABAs and LABAs [14].

### 2.2. Target 2: Muscarinic Receptors

**Figure 2:**
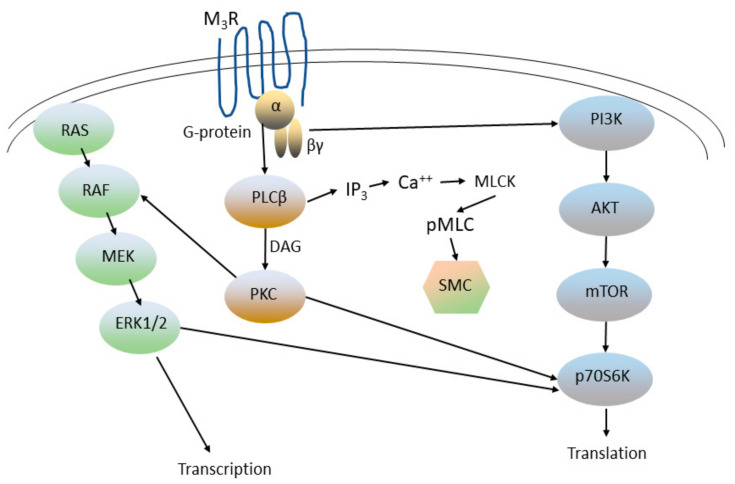
Signal transduction pathways downstream of the M_3_ muscarinic receptor in respiratory epithelium. Acetylcholine is the physiologic agonist of the M_3_ receptor, a GPCR, while SAMAs and LAMAs act as receptor antagonists. Agonist binding leads to association of the M_3_ receptor with its trimeric G-protein. The G-protein Gq α subunit stimulates phospholipase Cβ1 (PLCβ), leading to the generation of 1,4,5-inositol triphosphate (IP_3_). Acutely, IP_3_ stimulates the release of calcium (Ca^++^) from intracellular stores, most notably though the ryanodine receptor. Ca^++^ fluxes activate myosin light chain kinase (MLCK), which phosphorylates 20 kDa regulatory myosin light chain (MLC) in the smooth-muscle cell (SMC) contractile apparatus. Activation of PLC also produces diacylglycerol (DAG), which is the physiological activator of protein kinase C (PKC). Short-term activation of PKC inhibits myosin light chain phosphatase (not shown), which otherwise counteracts the effect of MLCK and thus augments SMC contraction. Long-term PKC activity promotes several growth-stimulatory pathways, most importantly RAS/RAF/MEK/ERK1/2. Agonist binding of the M_3_ receptor also leads to dissociation of the βγ subunits of Gq, which bind to, and stimulate, IP_3_ kinase (PI3K), in turn activating a growth-stimulatory pathway through AKT/mTOR/p70S6K.

The M_2_ and M_3_ muscarinic receptors are GPCRs expressed on airway smooth muscle cells and on neurons that modulate their contractility, as well as respiratory epithelial cells and glandular (goblet) cells responsible for the production of mucus [8,15]. Inhaled muscarinic receptor antagonists are separated into two groups based on their pharmacologic half-life: Short-acting muscarinic antagonists (SAMAs), such as ipratropium, are administered every 4 to 6 h (or on an as-needed basis), while long-acting muscarinic antagonists (LAMAs), such as tiotropium, umeclidinium, and glycopyrrolate, are administered once or twice daily [5,6,16]. Acetylcholine binding to M_3_ receptors on airway smooth muscle cells causes them to become engaged with the trimeric G-protein G_q_, which activates phospholipase Cβ1 (PLCβ), thereby releasing inositol 1,4,5-trisphosphate (IP_3_; Figure 2, Refs. [8,17,18,19,20]). Signaling mediated by IP_3_, and also by several other signal transduction pathways mediated by M_3_ receptors, activates Ca^++^-mediated signaling, leading to phosphorylation of regulatory myosin light chain (MLC) in the contractile apparatus, an obligatory event for the induction of smooth muscle contraction [15]. M_3_ antagonists therefore block the action of acetylcholine at the M_3_ receptor, thereby leading to smooth muscle relaxation. M_2_ receptors, which are located on both neurons and on airway smooth muscle, have been implicated in vagally induced bronchoconstriction, and appear to be dysfunctional in asthma, but play an uncertain role in COPD [15]. Both M_2_ and M_3_ receptors play important roles in regulating mTOR and Ras/RAF/ERK pathways, critical to mesenchymal cell growth and differentiation and thereby critical to airway remodeling in response to inflammation [8,15].

Given that airway obstruction in COPD is not reversible with acute administration of bronchodilators, at least as measured by spirometry, the beneficial effects of β_2_-adrenergic agonists and muscarinic antagonists in COPD might be perceived as being somewhat surprising. However, both classes of these agents have numerous cellular functions in the lung beyond any acute effect on airway smooth muscle tone. Potential beneficial effects on these drugs in COPD are likely to include modulation of ion channel activity, effects on inflammation, and long-term effects on airway remodeling [1]. Although SABAs and LABAs are of modest clinical value in most patients with COPD, they are important therapies in those with co-existing asthma [1,2].

#### Regulation of Airway Smooth Muscle Tone by Other Molecular Targets

There are numerous pathways, other than muscarinic or β_2_-adrenergic receptor signaling, that are essential for regulation of airway smooth muscle tone. These include the effects of numerous ion channels, several GPCRs (other than the β_2_-adrenergic receptor or muscarinic receptors), receptors for cytokines and growth factors, as well as important interactions with neighboring cells and the extracellular matrix [21,22]. All these regulatory elements are being studied as potential therapeutic targets [21]; their clinical validation remains an important future objective.

### 2.3. Target 3: The Glucocorticoid (Adrenal Corticosteroid) Receptor

Inhaled corticosteroids, such as beclomethasone, budesonide, and fluticasone, are the mainstay of management of the inflammatory component of COPD [1,4]. Corticosteroids diffuse easily through the plasma membrane and act intracellularly by binding specifically to the glucocorticoid receptor, a member of a larger family of receptors that bind steroid hormones and other small-molecule ligands. Binding of corticosteroids to the glucocorticoid receptor complex leads to translocation of this complex to the nucleus, where it binds to specific glucocorticoid-responsive elements in active chromatin, thereby regulating expression of an extensive cohort of genes, including those involved in inflammation, metabolism, and numerous other functions [23]. Therapeutically, inhaled corticosteroids target inflammation in the lung by virtue of their mode of delivery: they are rapidly distributed in the airways upon inhalation, allowing them to be delivered to the lungs at therapeutic concentrations, while achieving significantly lower drug levels systemically (i.e., outside of the lung) than would be the case if they were administered orally or parenterally. Nonetheless, inhaled corticosteroids are associated with a number of non-inflammatory side effects, such as hyperglycemia, osteoporosis, cataracts, skin thinning, and, in children, effects on growth that produce a reduction of adult height [1,4]. An increased risk of infections, such as oral candidiasis and pneumonia, is also seen with the use of these drugs [4]. Given the importance of inflammation in the pathogenesis of COPD, there is an urgent need for additional drugs that target inflammation in COPD without the limitations and side effects of corticosteroids.

### 2.4. Target 4: cAMP-Specific Cyclic Nucleotide Phosphodiesterases (PDE4s)

The cAMP-specific phosphodiesterases, or PDE4 enzymes, break down (hydrolyze) cAMP and thereby play an important role in regulating cAMP signaling pathways in cells (Figure 1; Ref. [24]). The PDE4s are members of the cyclic nucleotide phosphodiesterase superfamily, which consists of a total of 11 families, defined on the basis of their substrate specificity (cAMP, cGMP, or dual-specificity for both cAMP and cGMP) and their ability to be inhibited by pharmacologic inhibitors specific to each family; PDE3, PDE4, PDE7, and PDE8 are cAMP-selective, while PDE1, PDE2, PDE10, and PDE11 are dual-specific [25,26]. To date, PDE4 and PDE3 have been shown to be pharmacologically relevant targets in COPD; the pharmacologic potential of other families in COPD has yet to be determined.

#### 2.4.1. Roflumilast

Several highly selective PDE4 inhibitors are in widespread clinical use, including roflumilast, which is administered orally for the treatment of COPD. Roflumilast and other PDE4-selective inhibitors elevate cAMP levels in cells, activate PKA, stimulate CREB phosphorylation (Figure 1), and thereby produce changes in gene activity that appear to be responsible for the anti-inflammatory and immunomodulatory effects of these drugs [24,27]. Given the ability of roflumilast to elevate cAMP levels in cells, and the abundant evidence for the function of PDE4 in the physiology of normal respiratory epithelium and in disease states [24,27,28], it would appear to have the potential to act as a bronchodilator synergistically with other cAMP-elevating agents, such as β_2_-adrenergic agonists (Figure 1). However, extensive clinical study of roflumilast and several other PDE4-selective inhibitors, such as cilomilast, has failed to demonstrate significant acute bronchodilator activity (reviewed in Refs. [1,4]). Clinical studies of roflumilast have shown it to reduce the frequency of COPD exacerbations and it may be of particular value in patients with severe COPD who have chronic bronchitis and/or are repeatedly hospitalized for COPD [29,30,31,32,33]. It also appears to be of value in patients who cannot tolerate inhaled corticosteroids or who are not well-controlled on inhaled LABA/LAMA/steroid combinations [1,32]. Nausea, diarrhea, and changes in mood and behavior, all of which are typical class side effects of oral PDE4 inhibitors, are potential limitations in its use [4,31].

#### 2.4.2. Tanimilast

The systemic side effects of roflumilast and other oral PDE4-selective inhibitors have provided an impetus for the development of PDE4-selective inhibitors that could be administered by inhalation. These efforts led to the development of tanimilast (formerly known as CHF6001), a PDE4-selective inhibitor administered by inhalation [34]. A single phase 3 clinical trial in patients with COPD showed tanimilast to be well tolerated, with gastrointestinal side effects that were indistinguishable from those of the placebo [35]. Several ongoing phase 3 clinical trials (all listed on www.clinicaltrials.gov, as of 30 October 2023) are evaluating tanimilast in COPD, with special emphasis on patients with co-existing bronchitis and/or eosinophilia and on patients who are doing poorly on inhaled SAMA/LAMA and/or inhaled corticosteroid therapy. At least one of the ongoing trials is comparing tanimilast to roflumilast in patients with COPD using an active-comparator design. The results of these clinical trials, which are eagerly awaited, should provide critical proof-of-principle of the value of inhaled PDE4-selective inhibitors in COPD and possibly in other lung diseases.

#### 2.4.3. Ensifentrine

Ensifentrine (formerly known as RPL554) is an inhaled (nebulized) PDE inhibitor that is in late-stage clinical trials for the treatment of COPD. A similar drug, TQC3721, is being evaluated in clinical trials in China. Pharmacologically, ensifentrine is a dual inhibitor of both PDE3, the cGMP-inhibited cAMP-selective PDE, and PDE4. Although it has greater potency for PDE3 in pre-clinical assays, clinically, it appears to act predominantly as an inhibitor of PDE4 [36,37,38]. Unlike roflumilast, it appears to have significant bronchodilator activity [39]. It appears to be of value in improving symptoms and preventing hospitalizations in patients with moderate to severe COPD who require additional intervention despite optimal therapy with a LABA or LAMA [40]. Its value in patients who require inhaled corticosteroids appears promising but may require additional study. Largely because of its inhaled mode of administration, the side effects of ensifentrine are essentially indistinguishable from placebo [40]. The successful development of ensifentrine, which will be available for routine clinical use in the relatively near future, provides additional validation of PDE3/4-selective inhibitors in COPD and provides justification for the development of PDE3/4 inhibitors that can be used in a metered-dose inhaler or similar device.

## 3. Emerging Targets in COPD Therapeutics

### 3.1. Target 5: The Interleukin-4/Interleukin-13 Receptor

**Figure 3:**
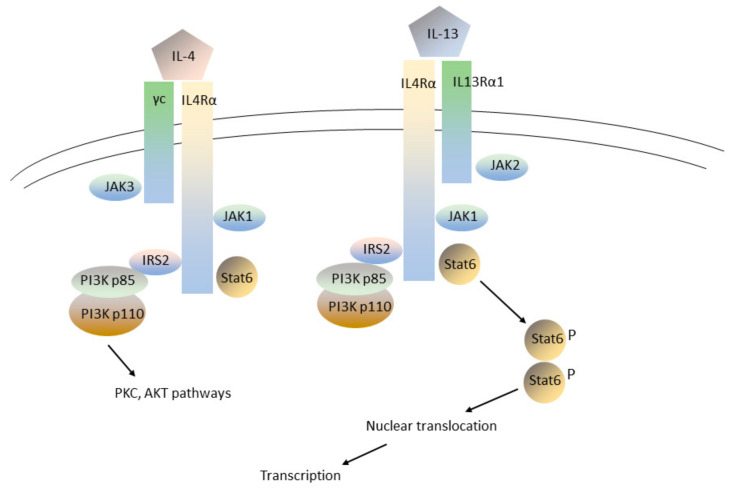
IL-4 and IL-13 share a common receptor subunit and activate common downstream signaling pathways in immune and other cells. The IL-4 receptor is a heterodimer, consisting of an alpha chain (IL4Rα) and a gamma (γc) subunit common to other cytokine receptors. The IL-13 receptor is also heterodimeric, consisting of IL4Rα and IL13Rα1. IL4Rα binds to the Janus tyrosine kinase JAK1, while the γc portion of the IL-4 receptor and IL13Rα1 bind to JAK3 and JAK2, respectively. Ligand binding by IL-4 or IL-13 activates JAK kinase activity, causing phosphorylation of IL4Rα at specific tyrosines that greatly increase its affinity for several adaptor proteins. The adaptor protein IRS2 recruits the p85 subunit of PI3K, which in turn binds the PI3K p110 subunit and activates PI3K. PI3K activation in turn stimulates the AKT and PKC pathways. JAK phosphorylation of IL4Rα also leads to the recruitment and tyrosine phosphorylation (P) of several Stat isoforms, most notably Stat6, which then homodimerize, translocate to the nucleus, and activate transcription of multiple genes involved in growth regulation and immunity/inflammation.

Given the importance of inflammation to the pathogenesis of COPD, the search for drugs that target immune/inflammatory pathways has been a major emphasis of recent and ongoing COPD drug development. However, until very recently, no anti-inflammatory drug, other than inhaled corticosteroids and roflumilast, had been shown to produce substantial clinical benefit in patients with COPD [1,4]. This situation was in distinct contrast to recent drug development in other immune/inflammatory states, such as rheumatoid arthritis and inflammatory bowel disease, where a large number of new therapeutic entities, especially monoclonal antibodies targeting various key regulators of the immune system, have become available for routine clinical use [41,42]. In particular, clinical trials of mepolizumab and benralizumab, which target interleukin-5 and which show substantial clinical activity in asthma, have shown insufficient clinical activity in clinical trials in COPD to justify clinical use [43,44]. However, a recent landmark phase 3 clinical trial has shown that dupilumab, a fully human monoclonal antibody that blocks the subunit in common to both interleukin-4 (IL-4) and interleukin-13 (IL-13) receptors (Figure 3; Refs. [45,46]), has substantial clinical activity in a specific subset of patients with COPD (Ref. [47]; a second phase 3 trial has been reported only as a press release).

In describing the clinical benefit of dupilumab, it is important to distinguish between various types of cell-mediated effector immunity, with specific relevance to inflammation in the lung. Type 1 immunity is characterized by CD4+ T_H_1 cells that secrete interleukin-2, interferon-γ, and lymphotoxin-α, is triggered by intracellular bacterial infections, and is characterized by phagocyte activation [48,49]. Type 2 immunity is characterized by CD4+ T_H_2 cells that secrete interleukins-4, -5, and -13, is triggered by parasitic infections, and is characterized by high antibody levels and eosinophilia [48,49]. Disordered Type 2 immune responses are associated with atopic diseases, such as allergy and asthma [50]. Type 3 immunity is characterized by CD4+ T_H_17 cells that secrete interleukin-17 and/or interleukin-22, and is triggered by extracellular bacterial and fungal pathogens [48,51,52]. Dupilumab is especially effective in Type 2 immunity because it targets the subunit common to both IL-4 and IL-13 receptors (Ref. [53], Figure 3). In contrast, mepolizumab and benralizumab target only interleukin-5 (Refs. [43,44]).

The phase 3 clinical trial that demonstrated the benefit of dupilumab in COPD was limited to patients who were on triple therapy with a LAMA, LABA, and an inhaled corticosteroid (or had contra-indications to inhaled corticosteroids), who had symptoms of bronchitis, and had an absolute blood eosinophilia of greater than 300 per microliter [47]. Retrospective analysis of patient data, including that from a number of clinical trials, has implicated eosinophilia as a predictor of response to corticosteroid therapy in COPD [4,54,55,56,57,58]. Eosinophilia also has predictive/prognostic value in COPD that appears to be independent of spirometric determinations of airway capacity [58]. In the design of the dupilumab clinical trial, eosinophilia was felt to select for a subset of COPD patients with dysfunctional Type 2 immunity who would be most likely to benefit from the drug [47]. The successful application of dupilumab in COPD should encourage the development or application of other agents targeting Type 2 immunity in COPD, as described in more detail below (see Implications for Drug Discovery).

#### Effects of IL-4 and IL-13 on Airway Mucus Production

In addition to their effects on Type 2 immunity in COPD, IL-4 and IL-13 signaling also play essential roles in mucus secretion by airway goblet and epithelial cells (see Ref. [59] for a general review of mucus in lung function in health and disease). Airway mucus is an essential component of airway defense, as demonstrated most definitively by the profound susceptibility to various bacterial pathogens, including *Staphylococcus aureus*, in mice with homozygous knockouts of the mucin glycoprotein gene *Muc5b* [60]. IL-4 and IL-13 are essential for allergen-induced goblet cell metaplasia in allergen-sensitized mice [61,62,63,64], and activation of Stat6-mediated gene transcription (Figure 3; Ref. [65]) is essential for the expression of mucin glycoprotein genes. Collectively, these data implicate an important link between immune activation pathways and airway mucus production and provide further experimental validation of the role of IL-4 and IL-13 signaling in COPD.

### 3.2. Target 6: IL-33 Signaling

Interleukin-33 (IL-33) is a cytokine that is released from epithelial cells, and probably other cell types, following injury or cell necrosis [52,66]. It in turn binds to its specific receptor, Il1rl1, often called ST2, which is a member of the IL-1 cytokine receptor family and is present on mast cells, basophils, and eosinophils [67]. Binding of IL-33 to ST2 stimulates the release of several cytokines, most importantly IL-5 and IL-13, as well as mediators of smooth-muscle tone in both blood vessels and airways [52]. Therefore, the activation of Il1rl1/ST2 is a powerful stimulation of Type 2 cell-mediated effector immunity [52,67]. Population-based studies of gain-of-function genetic variants in the genes encoding IL-33 and Il1R1 have shown them to be predictive of increased COPD risk [68]. Three different monoclonal antibodies have been evaluated clinically in a variety of inflammatory disorders, including asthma, and are currently in phase 3 clinical trials for COPD (see www.clinicaltrials.gov; search conducted 30 October 2023). Itepekimab targets IL-33 and has shown significant activity in patients with asthma [69]. Itepekimab has been tested in a randomized, placebo-controlled phase 2a trial in patients with moderate/severe COPD who were on stable doses of a SAMA/LAMA; most patients were also receiving inhaled corticosteroids [68]. The results from this phase 2a trial have led to the initiation of two phase 3 trials of itepekimab in former smokers with COPD. Astegolimab targets Il1rl1/ST2 and has been tested in a randomized, placebo-controlled phase 2a trial in patients with moderate/very severe COPD who were on triple therapy with a SAMA/LAMA/inhaled corticosteroid [70]. Two phase 3 trials in COPD are ongoing. Finally, tozorakimab, which can block signaling mediated by both reduced IL-33 and oxidized IL-33 (see Ref. [71]), has been tested in patients hospitalized for COVID-19 pneumonia [72]; two phase 3 trials in COPD are ongoing. Successful completion of any of these clinical trials would provide essential verification of IL-33 and/or its receptor as key therapeutic targets in COPD.

### 3.3. Target 7: Ion Channels and Mucociliary Clearance

#### 3.3.1. Genetic Insights into COPD Pathogenesis

The genetics of COPD is an underutilized approach in the field of lung research. The 2023 report of the Global Initiative for Chronic Obstructive Lung Disease (GOLD) mentions only α-1 antitrypsin deficiency as a noteworthy genetic contribution to our understanding of COPD [1]. However, it has long been realized that individuals with cystic fibrosis (CF) develop lung disease that has many of the hallmarks of severe COPD: severe bronchitis, recurrent infections with unusual pathogens, such as *Pseudomonas aeruginosa*, and frequent respiratory exacerbations. The radiological and pathologic features of CF show many resemblances to those of severe bronchitis [73]. An important distinction between CF and typical COPD is that patients with CF do not benefit from therapy with β_2_-adrenergic agonists or muscarinic receptor antagonists unless they have concurrent asthma [2] or have experienced environmental exposure (air pollution, smoking) of the type that leads to typical COPD. However, given the similarities that do exist between CF and COPD, the study of potentially overlapping disease mechanisms can identify additional targets for therapy common to both disorders.

#### 3.3.2. CF as a Severe Form of Bronchitis/COPD

CF is defined genetically by bi-allelic loss-of-function mutations in CFTR [74]. CFTR is an ATP-gated, cAMP-stimulated, Cl^−^ ion channel located in the apical plasma membrane of respiratory epithelium, with particularly strong expression in pulmonary ionocytes, as well as physiologically important expression in airway ciliated epithelial cells and potentially in other cell types (Figure 1, Refs. [9,10,11,75]. Clinically important mutations in CFTR variously reduce or affect the regulation of CFTR Cl^−^ ion channel function, reduce the synthesis/stability of CFTR protein in cells, or block the trafficking of CFTR to the apical plasma membrane [76,77]. The physiological effect of these mutations is to reduce Cl^−^ ion channel activity in the respiratory epithelium, with reduced volume and reduced pH of airway surface liquid [73]. Normally, airway mucus production and stability require cAMP-dependent, CFTR-dependent Cl^−^ transport and, critically, transport of bicarbonate [78]. Altered CFTR Cl^−^ ion channel activity thereby alters pulmonary mucus, leading to profound impairment of mucociliary transport [73]. In patients with either CF or COPD, abnormalities in these processes increase susceptibility to lung infections by a variety of bacterial pathogens, most typically *Pseudomonas aeruginosa*, leading to airway inflammation and bronchiectasis [73]

#### 3.3.3. Acquired CFTR Dysfunction in COPD

There is emerging evidence that acquired (i.e., non-genetic) deficiencies in CFTR function are common in patients with COPD. Studies from several different groups have shown that acute treatment with cigarette smoke decreases the level and/or activity of CFTR in respiratory epithelium [79,80,81,82,83]. Although cigarette smoke has numerous deleterious effects on respiratory function, both short- and long-term [84], these studies are consistent with the concept that smoke exposure leads to the development of a CF-like phenotype in the lung that contributes to the pathogenesis of COPD. It is important to note that these abnormalities develop even in patients who are genotypically wild-type for CFTR (i.e., they do NOT have CF). These acquired deficiencies in CFTR function in COPD, which have been studied in several well-validated pre-clinical and clinical COPD models, show that there are significant similarities in pathogenesis between CF and COPD, not just similarities in symptoms. Therapeutically, these observations suggest that agents that augment CFTR action at the apical membrane of epithelial cells and ionocytes, even in patients who do not have CF, have the potential to ameliorate clinical aspects of COPD.

#### 3.3.4. Regulation of CFTR by PKA Phosphorylation in CF and COPD

Among the most important regulators of CFTR Cl^−^ ion channel activity is the phosphorylation of the CFTR regulatory (R) domain by PKA and potentially by other kinases (Figure 1, Refs. [75,85,86,87,88,89,90,91]). Within the R-domain are numerous serines, located within consensus PKA phosphorylation sites, which serve to target PKA action to physiologically relevant regions of the R-domain [92]. R-domain phosphorylation produces profound alterations in its conformation, which in turn relieve its inhibitory effect and thereby potentiate CFTR gating (Figure 1; Refs. [75,93,94,95,96]). PKA appears to be anchored at the apical plasma membrane, at or near CFTR, by its interaction with specific A-kinase anchoring proteins (AKAPs, Refs. [90,91]).

Given the importance of PKA-mediated R-domain phosphorylation to CFTR function, there should be considerable interest in the development of drugs that increase PKA activity at the apical plasma membrane and thereby augment PKA-mediated CFTR phosphorylation. Such drugs might have value in specific allelic CF variants [77], but would also act on wild-type CFTR and therefore be of benefit in COPD. Several groups, including our own, have shown that roflumilast and other PDE4-selective inhibitors block the hydrolysis of cAMP, elevate cAMP levels at the apical membrane, enhance PKA-mediated phosphorylation of the CFTR R-domain, and enhance CFTR Cl^−^ ion channel activity [27,97,98,99,100]. This augmentation of CFTR Cl^−^ ion channel activity has been shown to benefit airway surface liquid and to augment mucociliary clearance [99,101]. Consistent with this mechanism, one potential mechanism of smoke-induced CFTR dysfunction is upregulation of PDE4 activity, leading to lower cAMP levels, decreased PKA action, and lower CFTR activity [102,103]. Therefore, it has become increasingly apparent that many of the benefits of roflumilast in COPD may be mediated by its effects on CFTR Cl^−^ ion channel activity.

There is also evidence to support the role of PDEs other than PDE4 in CFTR regulation, and therefore as targets in the treatment of COPD. PDE3A has been localized to the actin cytoskeleton in the vicinity of CFTR, and disruption of the PDE3A-actin interaction blocks compartmentalized CFTR regulation by PDE3A [104,105]. Acute exposure to cigarette smoke has been shown to upregulate PDE3 activity, which would reduce CFTR R-domain phosphorylation and thereby attenuate CFTR ion channel activity [103]. Given that ensifentrine is a dual PDE3/PDE4 inhibitor, and that both of these PDEs play an important role in the regulation of CFTR, it is quite likely that the beneficial effects of ensifentrine in COPD may be caused, at least in part, by its effects on CFTR [106,107].

#### 3.3.5. Direct Modulation of CFTR Cl^−^ Ion Channel Activity in COPD

An alternative method for the augmentation of CFTR activity is the use of the small-molecule CFTR-selective agent ivacaftor (formerly known as VX-770). Ivacaftor, and its deuterated derivative deutivacaftor, act as potentiators of CFTR action, in that they increase channel gating of CFTR and thereby enhance Cl^−^ ion transport [108]. Ivacaftor has single-agent activity on wild-type CFTR and on an intriguing subset of CFTR mutations, most notably gating mutations, including G551D (Refs. [109,110,111,112]), and also on specific mutations that affect CFTR processing or function (Ref. [113]; see Ref. [77] for a review). Collectively, these mutations allow at least a portion of CFTR to localize appropriately at the apical plasma membrane and exhibit at least partial Cl^−^ ion channel activity; ivacaftor augments the Cl^−^ ion channel activity of wild-type CFTR, and increases residual Cl^−^ ion channel activity in these CFTR mutants, by acting at a specific site in the CFTR transmembrane region [114,115]. Ivacaftor activates CFTR in a phosphorylation-dependent, but ATP-independent, manner [111,116]; it does not influence the phosphorylation status of the CFTR R-domain [117]. It has beneficial effects on mucociliary transport and in rescuing CFTR inhibition by acute exposure to smoke [118,119]. Ivacaftor has been tested in clinical trials in patients with bronchitis (i.e., with wild-type CFTR; Ref. [120]), but was subsequently withdrawn by the manufacturer from further clinical study in COPD. Icenticaftor (formerly known as QBW251) appears to work in a manner similar to that of ivacaftor, although the location/mechanism of its binding to CFTR has yet to be determined [121]. It has been studied in several early-phase clinical trials in COPD (i.e., in patients with wild-type CFTR), but currently is not undergoing clinical evaluation [122,123]. Although the clinical trial data on current CFTR potentiators has yet to lead to clinical use of these agents in COPD, the data to date confirm the concept that targeting CFTR Cl^−^ ion channel function has the potential to produce meaningful improvements in patients with COPD.

## 4. Implications for Clinical Care, Clinical Trials and Drug Discovery

Key concepts:

- Defining a “precision medicine” approach to the care of patients with COPD. For this purpose, they can be classified into one of several distinct groups:
  ◦ Patients with predominantly manifestations of bronchitis
  ◦ Patients with eosinophilia (and potentially other biomarkers of Type 2 inflammation)
  ◦ Patients without bronchitis
- Designing the next generation of clinical trials, to reflect emerging standards in COPD patient care and research:
  ◦ Improved patient selection (inclusion/exclusion criteria and/or patient stratification), ideally reflecting the classification outlined above
  ◦ The use of biomarkers as a patient selection tool, and as intermediate endpoints in early-phase clinical trials
  ◦ Improved clinical trial design that controls for concomitant medications and ensures that patients are on stable doses of concomitant medications prior to initiation of protocol therapy
  ◦ Active-comparator design

### 4.1. Implications for Clinical Care

The expanding range of therapeutic targets in COPD makes it possible, indeed necessary, to define specific subsets of patients with COPD. Such a classification system would reflect our growing knowledge of the pathophysiology of COPD and would in turn introduce a “precision medicine” approach that would inform translational research, clinical trials, and patient care. Various COPD classification systems have been proposed [1], but to date, none have been based on the approach of assessing the relative value of specific drug targets to the study and care of any individual patient. The studies summarized in this review have shown that the selection of drug targets in an individual patient has become increasingly precise and evidence-based, reflecting the increasing number of drugs available for patients with COPD. Here, I suggest a tentative classification of COPD based on the targets most likely to be of value to specific sub-groups of patients. This classification system is applicable primarily to older adults; other classification systems have been applied to other populations [1].

#### 4.1.1. Patients with Predominant Manifestations of Bronchitis

This group of patients has airway obstruction with significant bronchitic symptoms. They clearly have evidence of active airway inflammation, without peripheral blood eosinophilia. Many of these patients will be actively exposed to cigarette or environmental smoke and indeed their clinical presentation may resemble tobacco-exposed persons with symptoms and preserved lung function (who technically do not have COPD [124]). Patients in this group generally benefit from inhaled β_2_-adrenergic agonists and muscarinic antagonists. They also generally benefit from the anti-inflammatory and CFTR-activating action of roflumilast (and, in the future, will benefit from inhaled PDE4 inhibitors, such as tanimilast, and/or PDE3/4 inhibitors, such as ensifentrine).

#### 4.1.2. Patients with Eosinophilia

This group of patients is characterized by airway obstruction accompanied by abnormalities in Type 2 immunity, with active airway inflammation and eosinophilia. In addition to eosinophilia, these patients will have biomarkers of inflammation, such as an elevated FENO level [125] and elevated markers typical for disordered Type 2 immunity, such as Eotaxin-3, PARC, and IgE [47]. Many will have bronchitis, but bronchitis need not be a defining criterion for this patient group. Most of these patients would be treated with inhaled β_2_-adrenergic agonists and muscarinic antagonists. However, the presence of eosinophilia in these patients would predict that they would derive special benefit from inhaled corticosteroids and dupilumab. They will probably also benefit from roflumilast (and, in the future, inhaled PDE4 and PDE3/4 inhibitors), although eosinophilia has not been prospectively tested as a predictor of response to these drugs.

#### 4.1.3. Patients without Bronchitis

This large group of COPD patients is characterized by airway obstruction without clinical or routine laboratory evidence for inflammation. They are likely to be former or never smokers, rather than current smokers, and tend to have a more stable clinical course. However, many will have extensive emphysema on lung imaging. Most of these patients will be treated with inhaled muscarinic antagonists and may indeed specifically benefit from the airway remodeling effects of these agents. Most will also receive therapy with β_2_-adrenergic agonists; however, in the absence of asthma, they may not receive much benefit from this modality of therapy. Given the absence of bronchitis or eosinophilia, they will be less likely to benefit from roflumilast, corticosteroids, or dupilumab.

The COPD subsets described here are intended to guide the treatment selections for chronic therapy in stable patients, with the goals of improving symptoms and quality of life and in reducing exacerbations. Non-pharmacologic interventions, such as smoking cessation, reduction of environmental exposures, oxygen therapy, and rehabilitation, are also extremely important and have been described elsewhere [1]. The role of effective vaccines against pneumococcus, COVID-19, and RSV in the prevention of exacerbations has also been the subject of recent reviews [1]. Treatment of exacerbations, and potential classifications of COPD patients on the basis of the type of exacerbation [33,126], are the subject of extensive review elsewhere [1].

### 4.2. Implications for Clinical Trial Design

Our increasing appreciation of the diversity of patients with COPD, and the expanding number of therapeutic targets in the treatment of this disease, has important implications for the design of future clinical trials in persons with COPD. The next generation of clinical trials will need to address a number of key issues.

#### 4.2.1. Patient Selection and Stratification

Traditionally, clinical trials of interventions in COPD have adopted an “all-comers” approach to patient selection, using as clinical trial inclusion criteria of all patients with COPD whose disease met or exceeded a defined standard of severity (e.g., moderate-to-severe [30,32,127], mild to moderate [128], etc.; these standards have been codified recently by GOLD [1] and may be subtly different from those used in older clinical trials). However, given that most interventions are likely to have greater benefit in specific patient populations defined by clinical and/or laboratory criteria, the next generation of clinical trials will benefit from a precision medicine approach, where eligibility is targeted prospectively to specific subsets of persons with COPD. The recent dupilumab trial is an excellent example of this approach, where eligibility was limited to persons with COPD and dysfunctional Type 2 immunity, as defined by eosinophilia [47] (see Target 5, third paragraph for more details).

#### 4.2.2. Increasing the Role of Biomarkers

As our knowledge of the basic biology of COPD continues to evolve, it will be possible to use a biomarker approach to aid in the selection and stratification of patients on clinical trials. Early-stage clinical trials will also be able to use biomarkers as end-points, with a greater degree of confidence that these biomarkers will indeed be predictive of clinically meaningful endpoints in later-stage trials. Examples of biomarkers that can be used in these approaches include the eosinophil level, as described previously (see Target 5, third paragraph). Other examples of this approach might include the use of elevated markers typical for disordered Type 2 immunity, such as Eotaxin-3, PARC, and IgE; these biomarkers were recently validated in the dupilumab trial [47]. Another important clinical trial tool might be an elevated FENO level [125], a well-validated general biomarker for inflammation [125].

#### 4.2.3. Concurrent Therapy

As therapies multiply, there will be an increasing heterogeneity of patients being assessed for clinical trials on the basis of concurrent and/or recent past treatment with various COPD medications. Given this evolution in the clinical trial space, several issues emerge:

(a) Publications of placebo-controlled trials need to report all data on concurrent medications. In the recent dupilumab trial, all patients were receiving triple LABA/LAMA and inhaled glucocorticoid therapy (or had contra-indications to glucocorticoid therapy), but the proportion, if any, of patients who were taking roflumilast, or other COPD-modifying drugs, was not specified [47]. These data are essential to allow clinicians to determine the applicability of the trial to “real world” situations: i.e., to determine which patients in routine clinical care would be appropriate candidates for the therapy studied in the trial.
(b) Future trials will need to use concurrent COPD medications as criteria for inclusion/exclusion, or as a stratification tool (i.e., as a pre-hoc factor in the randomization process).
(c) Clinical trialists will also need to ensure that patients are on optimal therapy with generally approved COPD medications and that they are on stable doses of these medications before being entered in a clinical study. Otherwise, improvement in a disease variable after the initiation of the drug (or placebo) in the trial might actually be caused by recent changes in the concurrent medication, rather than the drug being studied.

#### 4.2.4. Active-Comparator Trial Design

Given the increasing number of therapies active in COPD, there will be an increasing need to utilize clinical trials with an active-comparator design. Older trials, including the pivotal clinical trials of roflumilast, tiotropium, and dupilumab, used a placebo-controlled design [30,32,47,127,128] in which the intervention of interest was compared to an inactive placebo. Future definitive clinical trials are more likely to include an active comparator in which the therapy of interest is compared to a different agent, quite likely one of a different therapeutic class. Even today, the field of COPD therapeutics would benefit from clinical trial data comparing different classes of currently available agents. For example, clinical trials that compared inhaled corticosteroids to roflumilast, or roflumilast to dupilumab, with appropriate eligibility criteria none of which are currently in progress, would certainly benefit patient care.

## 5. Implications for Future Drug Discovery Strategies

Key concepts

- Targets involved in the regulation of cAMP
- Targets on the CFTR Cl^−^ ion channel
- Targets in the immune/inflammatory system
- Druggable targets essential for repair or regeneration of airway epithelium, airway smooth muscle and alveoli

Identification of agents with novel mechanisms of action remains critical to progress in COPD. Possible strategies for the identification of novel and effective drugs in COPD could include the following.

### 5.1. Novel Agents That Modulate cAMP Levels

In addition to β_2_-adrenergic receptors, PDE3s, and PDE4s, there are numerous other molecular components of cAMP signaling pathways in pulmonary tissue that have the potential to become therapeutic targets. Among GPCRs, these include the receptors for adenosine (the A_2A_, A_2B_ and A_3_ receptors), which act through G-proteins to regulate adenylyl cyclase [129,130,131,132,133], and the prostanoid receptor, which acts through a different G-protein to regulate adenylyl cyclase 2 (Ref. [134]). Among members of other PDE families, PDE8 isoforms have been implicated in the regulation of airway smooth muscle tone [135,136] and may be promising therapeutic targets in the future. Also worth mentioning in this section are the PDE5 enzymes, which hydrolyze cGMP (not cAMP) and are essential regulators of pulmonary arterial vasculature [137]. PDE5 inhibitors are important therapeutically in the treatment of primary pulmonary hypertension [138], although they have yet to be shown to have therapeutic value in patients with COPD who do not have primary pulmonary hypertension [139].

### 5.2. Novel CFTR Potentiators

The complex structure and regulation of CFTR provides a potential rich source of targets for drugs that augment CFTR function. For COPD, the focus would be on drugs that augment the function of un-mutated (wild-type) CFTR. These drugs could include agents that modulate CFTR R-domain phosphorylation, or, in the manner of currently available CFTR potentiators, interact directly with the CFTR protein to augment its Cl^−^ ion channel activity.

### 5.3. Novel Anti-Inflammatory or Immunomodulatory Therapies

There is an obvious need for additional therapies that target inflammation in COPD. These might target eosinophils directly, or alternatively, target downstream effector cells, such as activated macrophages or neutrophils. Agents that have been studied successfully in other immune/inflammatory disorders are obvious candidates for clinical trials in COPD. One choice might be lebrikizumab, which, like dupilumab, targets the Interleukin-4/Interleukin-13 receptor [140]. JAK inhibitors (Figure 3) may also be promising agents to study, in view of their proven action in several other inflammatory conditions.

### 5.4. Novel Agents Targeting Airway Remodeling

Irreversible airway loss is a hallmark of COPD, yet there are no therapies that directly target airway loss, or stimulate the growth of new airways, in this disease. Generation and the clinical study of agents that stimulate airway regeneration is an essential long-term objective of COPD research.

## 6. Conclusions

Identification of key molecular targets in COPD greatly illuminates therapeutic options in individual patients and is essential for precision medicine. Ongoing research in COPD biology and therapeutics should facilitate the identification of novel and emerging targets, such as ion channels, cytokines and their receptors, and growth factors implicated in airway remodeling. Precise identification of targets will also streamline the design of clinical trials and enhance the effectiveness of pre-clinical drug discovery strategies.

## Data Availability

Data sharing not applicable. No new data were created or analyzed in this study. Data sharing is not applicable to this article.
